# Inebriate Asylums

**Published:** 1888-12

**Authors:** 


### Editorial.

### INEBRIATE ASYLUMS.

We notice that for the third time the subject of an inebriate asylum for the State of Georgia has been brought to the attention of the Legislature of Georgia. In response to the action of the Legislature at its last session, a report has been presented through the Governor to that body, that ought to have more than usual force, from the fact that the Superintendent of the Lunatic Asylum of the State (where a number of inebriates have been sent for treatment) was a member of the committee designated by the Legislature to investigate and report upon the subject, and are in full accord with the action of the committee in presenting a convincing argument and testimony in behalf of the establishment of such an institution. The truth is that the importance of such institutions has been so fully established by actual demonstration, and the results, as exhibited in the various reports from these institutions at the North, have been so eminently decisive as to their beneficent effects in the restoration of inebriates to sobriety and usefulness, their value cannot longer be considered an open question. The necessity for an institution of this character in the State of Georgia, and the good that may be accomplished by its establishment being considered as settled, the real question at issue is whether the Legislature can be brought to the appreciation of the obligation resting upon that body and respond to the solemn duty involved in this matter. It would seem to us that independent of other conclusive considerations which should urge that body to meet this duty in some decided way, which have been so fully and intelligently presented by the three reports referred to, our legislators should be impelled to an effort to relieve the State of the drunkenness, crime, insanity and destitution so apparent and wide-spread as the immediate and palpable result of the legalized traffic in alcoholic liquors, for which the State government is directly responsible. Will anything be done commensurate with the vital interest involved?

